# Effect of the Chloro-Substitution on Electrochemical and Optical Properties of New Carbazole Dyes

**DOI:** 10.3390/ma14113091

**Published:** 2021-06-04

**Authors:** Przemysław Krawczyk, Beata Jędrzejewska, Klaudia Seklecka, Joanna Cytarska, Krzysztof Z. Łączkowski

**Affiliations:** 1 Department of Physical Chemistry, Faculty of Pharmacy, Collegium Medicum, Nicolaus Copernicus University, Kurpińskiego 5, 85-950 Bydgoszcz, Poland; 2 Faculty of Chemical Technology and Engineering, UTP University of Science and Technology, Seminaryjna 3, 85-326 Bydgoszcz, Poland; beata@utp.edu.pl; 3 Department of Chemical Technology and Pharmaceuticals, Faculty of Pharmacy, Collegium Medicum, Nicolaus Copernicus University, Jurasza 2, 85-089 Bydgoszcz, Poland; imaginowa@o2.pl (K.S.); cytar@cm.umk.pl (J.C.); krzysztof.laczkowski@cm.umk.pl (K.Z.Ł.)

**Keywords:** carbazole dyes, optical functional materials, photo-optical properties, nonlinear optical properties, toxicology, bioimaging, protein affinity, DFT

## Abstract

Carbazole derivatives are the structural key of many biologically active substances, including naturally occurring and synthetic ones. Three novel (E)-2-(2-(4-9*H*-carbazol-9-yl)benzylidene)hydrazinyl)triazole dyes were synthesized with different numbers of chlorine substituents attached at different locations. The presented research has shown the influence of the number and position of attachment of chlorine substituents on electrochemical, optical, nonlinear, and biological properties. The study also included the analysis of the use of the presented derivatives as potential fluorescent probes for in vivo and in vitro tests. Quantum-chemical calculations complement the conducted experiments.

## 1. Introduction

Carbazole, naturally occurring in coal tar, is a tricyclic molecule with the carbon skeleton of fluorene. Intensive research into carbazole derivatives began in 1872 with the description by Graebe and Glaser of the 9*H*-carbazole [1]. In turn, research on their pharmacological properties began with the discovery of antibacterial activity of murrayanine extracted from the stem bark of Murraya Koenigii (Rutaceae) led to the discovery of new pharmacological activities [2,3]. The rapid development of work on this class of compounds has shown that they are characterized by excellent photo-conductivity with high luminescent efficiency. The displayed fluorescent properties make this class of compounds a molecular element of many compounds used for the production of dyes [4,5,6], polymers [7,8,9], or electroluminescent materials [10,11,12,13,14] and are applicable as organic light emitting diodes (OLEDs) [15,16,17,18,19], thin film transistors, photorefractive materials [20], and sensors [21,22]. Carbazole derivatives are also a building block of the D-A conjugated polymers used in the nonlinear optical maters. Due to their good solubility, they are widely used in production of organic photoconductors and photorefractive materials in OLEDs [23]. Carbazole is characterized by high structural rigidity and high thermal stability [24]. In addition, it has good hole transporting properties [25,26,27], and due to its intense blue luminescence, it is used as a building block for the production of various optoelectronic devices [28,29,30]. Carbazoles are an attractive structural motif because of the multiple sites of potential functional group attachment. For example, derivatives with attached chromophores through C-2, C-3, C-6, C-7, and N-9 are used in in organic light-emitting diodes, photovoltaic devices [31], solar cells [32,33], design of organic semiconductors, adsorbent for organic dyes [34], and inter alia [35].

Earlier studies suggest that the combination of a thiazole system with a carbazole ring significantly enhances the hyperpolarizability of dyes [36]. Active carbazole alkaloids exhibit many interesting biological activities, which led many scientists to address structural modifications of naturally occurring derivatives. The second common interest in the world of science is the synthesis of new derivatives with desired chemical and biological properties. Many biologically active of carbazole derivatives (alkaloids) of natural origin, as well as products of the synthesis of drugs characterized by anti-HIV, anti-cancer, antibacterial [37], antifungal [38], antioxidant [39], hepatoprotective, antiprotozoan, anti-inflammatory, and sedative properties, as well as topoisomerase II inhibition ability [40,41,42] and pancreatic cholesterol esterase inhibitory activity [43]. Many compounds of this class have a very high activity against many organisms, namely bacteria, fungi, parasites [44,45,46,47,48,49,50], or are potential anti-inflammatory agents [51]. Many of them are potential multifunctional agents for the treatment of neurological disorders [52,53,54,55], characterized by anticancer activity [56,57,58,59,60,61,62,63], or even anti-SARS-CoV-2 drugs [64]. In addition, molecules belonging to carbazole derivatives can noncovalently interact with DNA by electrostatic interactions, through intercalation and the minor groove binding. This causes that they are also tested as new pharmacologically active substances and molecules of biological importance.

Keeping this in mind, we decided to design and then synthesize three carbazole-based thiazoles, namely 2-(2-(4-(9*H*-carbazol-9-yl)benzylidene)hydrazinyl)-4-(4-chlorophenyl) thiazole (P1), 2-(2-(4-(9*H*-carbazol-9-yl)benzylidene)hydrazinyl)-4-(2,4-dichlorophenyl) thiazole (P2), and 2-(2-(4-(9*H*-carbazol-9-yl)benzylidene)hydrazinyl)-4-(3,4-dichlorophenyl) thiazole (P3). The study presents the synthesis, spectroscopic characteristics, nonlinear optical properties (NLO), and biological activities of the mentioned compounds. The work also includes toxicological analysis and two-photon absorption cross section (TPA) calculations. The main aim of this work is to evaluate the effect of the number and point of attachment of chlorine substituents on the physicochemical, optical, and biological properties. In addition, the presented research is an introduction to a wider issue related to the development of new fluorescent markers in living cell imaging technology and flow cytometry. Moreover, due to the low calculation cost and high precision in reproducing the measured values, the research was supported by theoretical calculations using density functional theory (DFT).

## 2. Methodology Section

### 2.1. Chemical Synthesis

All substrates used within the present study were best quality commercial materials by Aldrich, and were used without purification. Syntheses were carried out in air atmosphere. The ^1^H (700 MHz) and ^13^C (176 MHz) NMR spectra were measured using Bruker Avance III spectrometer. The ESI-HRMS analyses were carried out in the Laboratory for Analysis of Organic Compounds and Polymers at the Polish Academy of Science in Łódź. Open glass capillaries were used to determine melting point values for all synthesized products. Macherey-Nagel Polygram Sil G/UV254 0.2 mm plates were used in TLC analysis.

**Scheme 1:**
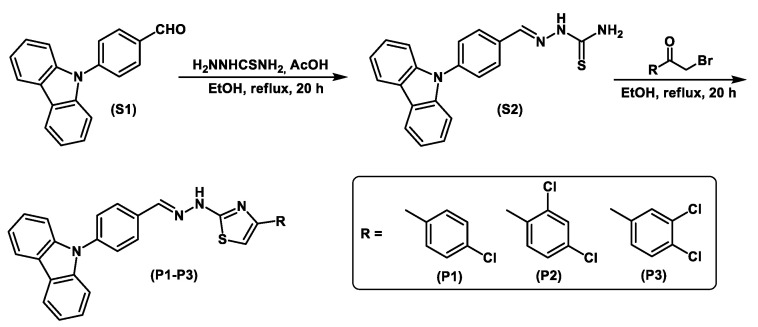
Synthesis of the target compounds P1–P3.

A two-step reaction was used to obtain carbazole-thiazole derivatives P1–P3 containing chlorine substituents in the phenyl ring (Scheme 1). As the first step, the 4-(9*H*-carbazol-9-yl)benzaldehyde S1 was reacted with thiosemicarbazide in ethanol under reflux to produce the corresponding 2-(4-(9*H*-carbazol-9-yl)benzylidene)hydrazinecarbothioamide S2 in high yield of 90%. In the second step, hydrazinecarbothioamide S2 reacts with the appropriate 2-bromo-1-(4-chlorophenyl)ethanone, 2-bromo-1-(2,4-dichlorophenyl)ethanone, and 2-bromo-1-(3,4-dichloro-phenyl)ethanone in ethyl alcohol and alcohol under reflux to produce target products with 84–99% yield and high chemical purity. Silica gel column chromatography was used for all compounds purification. Products were next characterized using spectroscopic methods: ^1^H, ^13^C NMR, and ESI-HRMS. In the NMR spectra of the products P1–P3, broadened hydrazine NH singlet at 12.32–12.35 ppm, and a characteristic singlet at 8.18 ppm corresponding to the CH=N group were observed. The signals of carbon atoms in C=N and C-NH groups appear in the ^13^C NMR spectrum around 149 and 169 ppm. Moreover, mass spectra of the products P1–P3 showed peaks corresponding to their molecular [M+H]^+^ ions. 

#### 2.1.1. 2-(4-(9H-Carbazol-9-yl)benzylidene)hydrazinecarbothioamide (S2)

Thiosemicarbazide (0.91 g, 10.0 mmol) and next (0.8 mL) of acetic acid were added to a stirred solution of 4-(9*H*-carbazol-9-yl)benzaldehyde (S1) (2.71 g, 10.0 mmol) in absolute ethyl alcohol (30 mL). The reaction mixture was stirred under reflux for 20 h under air atmosphere, then added to water (100 mL) and neutralized with NaHCO_3_ solution. The resulting solid was filtered off and washed with a water giving product with 3.10 g (90%) yield; mp 202–204 °C; eluent: dichloromethane/methanol (95:5), R_f_ = 0.92. ^1^H NMR (700 MHz, DMSO-d_6_), δ (ppm): 7.28–7.32 (m, 2H, 2CH); 7.42–7.46 (m, 4H, 4CH); 7.66 (d, 2H, 2CH, J = 7.7 Hz); 8.10–8.11 (m, 3H, 2CH, NH); 8.18 (s, 1H, CH); 8.25–8.26 (m, 3H, 2CH, NH); 11.53 (bs, 1H, NH). ^13^C NMR (176 MHz, DMSO-d_6_), δ (ppm): 110.18 (2C); 120.75 (2C); 121.02 (2C); 123.39 (2C); 126.81 (2C); 127.14 (2C); 129.44 (2C); 133.82 (C); 138.43 (C); 140.39 (2C); 141.81 (C); 178.74 (C).

#### 2.1.2. General Experimental Procedure for Synthesis of Derivatives (P1–P3)

Carbothioamide S2 (1.0 mmol) was added to a stirred solution of appropriate 2-bromoacetophenone (1.0 mmol) in absolute ethyl alcohol (20 mL). The reaction mixture was stirred under reflux for 20 h, then separated, added to water (50 mL), and neutralized with NaHCO_3_ solution. Silica gel column chromatography (230–400 mesh) was employed for product purification (eluent: dichloromethane/methanol (95:5)). 

#### 2.1.3. 2-(2-(4-(9H-Carbazol-9-yl)benzylidene)hydrazinyl)-4-(4-chlorophenyl)thiazole (P1) 

Yield: 0.49 g, >99%, (dichloromethane/methanol (95:5), R_f_ = 0.85); mp 240–244 °C. ^1^H NMR (700 MHz, DMSO-d_6_), δ (ppm): 7.29–7.33 (m, 2H, 2CH); 7.41–7.49 (m, 7H, 7CH); 7.70 (d, 2H, 2CH, J = 8.4 Hz); 7.88 (d, 2H, 2CH, J = 8.4 Hz); 7.95 (d, 2H, 2CH, J = 8.4 Hz); 8.18 (s, 1H, CH); 8.25 (d, 2H, 2CH, J = 7.7 Hz); 12.32 (bs, 1H, NH). ^13^C NMR (176 MHz, DMSO-d_6_), δ (ppm): 105.13 (C); 110.22 (2C); 121.01 (2C); 120.72 (2C); 123.43 (2C); 126.78 (2C); 127.35 (2C); 127.76 (2C); 128.38 (2C); 129.08 (2C); 130.03 (C); 132.51 (C); 133.92 (C); 138.00 (C); 140.44 (2C); 141.24 (C); 149.64 (C); 168.87 (C). ESI-HRMS (m/z) calculated for C_28_H_20_ClN_4_S: 479.1097 [M + H]^+^. Found: 479.1099 [M + H]^+^.

#### 2.1.4. 2-(2-(4-(9H-Carbazol-9-yl)benzylidene)hydrazinyl)-4-(2,4-dichlorophenyl)thiazole (P2)

Yield: 0.43 g, 84%, (dichloromethane/methanol (95:5), R_f_ = 0.87); mp 206–210 °C. ^1^H NMR (700 MHz, DMSO-d_6_), δ (ppm): 7.29–7.32 (m, 2H, 2CH); 7.43–7.48 (m, 5H, 5CH); 7.51–7.53 (m, 1H, CH); 7.70–7.73 (m, 3H, 3CH); 7.91 (d, 1H, CH, J = 9.1 Hz); 7.95 (d, 2H, CH, J = 8.4 Hz); 8.18 (s, 1H, CH); 8.26 (d, 2H, 2CH, J = 7.7 Hz); 12.33 (bs, 1H, NH). ^13^C NMR (176 MHz, DMSO-d_6_), δ (ppm): 109.83 (C); 110.21 (2C); 120.73 (2C); 120.99 (2C); 123.42 (2C); 126.79 (2C); 127.37 (2C); 127.94 (C); 128.38 (C); 130.21 (C); 132.15 (2C); 132.59 (C); 132.75 (C); 133.04 (C); 133.96 (C); 138.02 (C); 140.46 (2C); 141.26 (C); 146.38 (C); 167.93 (C). ESI-HRMS (m/z) calculated for C_28_H_19_Cl_2_N_4_S: 513.0707 [M + H]^+^. Found: 513.0712 [M + H]^+^.

#### 2.1.5. 2-(2-(4-(9H-Carbazol-9-yl)benzylidene)hydrazinyl)-4-(3,4-dichlorophenyl)thiazole (P3)

Yield: 0.49 g, 95%, (dichloromethane/methanol (95:5), R_f_ = 0.94); mp 219–220 °C. ^1^H NMR (700 MHz, DMSO-d_6_), δ (ppm): 7.29–7.33 (m, 2H, 2CH); 7.43–7.48 (m, 4H, 4CH); 7.59 (s, 1H, CH); 7.68 (d, 1H, CH, J = 8.4 Hz); 7.71 (d, 2H, 2CH, J = 7.0 Hz); 7.84–7.87 (m, 1H, CH,); 7.95 (d, 2H, 2CH, J = 8.4 Hz); 8.09–8.11 (m, 1H, CH); 8.18 (s, 1H, CH); 8.26 (d, 2H, 2CH, J = 8.4 Hz); 12.35 (bs, 1H, NH). ^13^C NMR (176 MHz, DMSO-d_6_), δ (ppm): 106.53 (C); 110.21 (2C); 120.72 (2C); 120.98 (2C); 123.42 (2C); 126.04 (2C); 126.78 (2C); 127.35 (C); 127.70 (C); 128.39 (2C); 130.23 (C); 131.30 (C); 131.95 (C); 133.93 (C); 135.73 (C); 138.04 (C); 140.45 (2C); 141.24 (C); 148.56 (C); 168.95 (C). ESI-HRMS (m/z) calculated for C_28_H_19_Cl_2_N_4_S: 513.0707 [M+H]^+^. Found: 513.0707 [M+H]^+^.

### 2.2. Experimental Measurements

#### Materials and Instruments

Solvents for spectroscopic measurements were obtained from Sigma-Aldrich Chemical Co. (St. Louis, MO, USA) or Alfa Aesar Co. (Waltham, MA, USA) They were spectroscopic grade and did not require further purification.

A Shimadzu UV-Vis Multispec-1501 (Kyoto, Japan) and a Hitachi F-7100 spectrophotometers (Hong Kong, China) were used for steady-state absorption and emission measurements, respectively. 

The fluorescence quantum yield of the dyes were determined according to Equation (1) [65]:

(1) $$\phi_{s}=\phi_{ref}\frac{I_{s}A_{ref}}{I_{ref}A_{s}}\cdot \frac{n_{s}^{2}}{n_{ref}^{2}}$$

where: *φ_ref_* is the fluorescence quantum yield of Coumarin 1 in ethanol (*φ_ref_* = 0.64 [49]), *A* is the absorbance at the excitation wavelengths (*A* ≈ 0.1 at 366 nm), *I* is the integrated emission intensity, *n* is the refractive index of the solvents used. Index “*s*” refers to the sample whereas “*ref*” to the reference.

An Edinburgh Instruments single-photon counting system (FLS920P Spectrometers) was applied to record fluorescence decay curves. The excitation of the sample was performed using a picosecond diode laser. It generates pulses of about 55 ps at 375 nm. The dyes were studied at dilute solution (0.2–0.3 in a 10 mm cell). The fluorescence lifetimes were determined by deconvolution of the fluorescence decays were using double-exponential functions. The average lifetime, *τ_av_* was calculated according to Equation (2):

(2) $$\tau_{av}=\frac{\sum \tau_{i}\alpha_{i}}{\sum \alpha_{i}}$$

where *α_i_* and *τ_i_* are the amplitudes and lifetimes, respectively.

### 2.3. Computational Details

Structural optimization of the analyzed compounds in their in their ground (*S_GS_*) and excited (*S_CT_*) states were performed using DFT approach implemented in Gaussian 09 program package [66] and PBE0/6-311++G(d,p) basis set. Based on the analysis of Hessians, it was confirmed that the obtained structures correspond to the minima on the potential energy surface, an analysis of Hessians was performed. The linear optical properties were obtained using the time-dependent density functional theory (TDDFT/PBE0) [67] and the state-specific (SS) corrected linear response (cLR) approach [68]. The PBE0 [69], CAM-B3LYP [70], HSEH1PBE [71,72], and LC-*ω*PBE [73,74,75] functionals were used in the simulation of spectroscopic parameters. In order to analyze the de-excitation energy, the ground state should be calculated with non-equilibrium solvation [76,77]. In the SS approximation, as opposed to TD-DFT, the solvent dynamic polarizations is determined by the difference of the electron densities of the initial and final states [68,78,79,80].

The dipole moments and polarities of the charge-transfer state (*CT*) were evaluated by numerical differentiation of the excitation energies (*E*) in the presence of an electric field *F* of 0.001 a.u. strength:

(3) $$\Delta \mu_{GS-CT}^{i}=\frac{E_{CT}\left(+F^{i}\right)-E_{CT}\left(-F^{i}\right)}{-2F^{i}}-\frac{E_{GS}\left(+F^{i}\right)-E_{GS}\left(-F^{i}\right)}{-2F^{i}}$$

where *i* stands for the Cartesian component of the dipole moment difference.

The polarizability anisotropy (Δ*α*), isotropic average polarizability (〈α〉), and first-order hyperpolarizability (*β_vec_*) were determined based on the Gaussian 09 program and defined as

(4) $$\langle \alpha \rangle =\frac{\alpha_{xx}+\alpha_{yy}+\alpha_{zz}}{3}$$

(5) $$\beta_{vec}=\underset{i=x,y,z}{\sum }\frac{\mu_{i}\beta_{i}}{\left|\mu \right|}$$

where $\beta_{i}\left(i=x,y,z\right)$ is given by $\beta_{i}=\left(\frac{1}{3}\right)\sum_{j=x,y,z}\left(\beta_{ijj}+\beta_{jij}+\beta_{jji}\right)$.

The charge transfer parameters, the amount of transferred charge (*q_CT_*), and the charge-transfer distance (*D_CT_*) have been determined following a Le Bahers’ procedure [81] using PBE/6-311G++G(d,p). 

The Integral Equation Formalism for the Polarizable Continuum Model (IEF–PCM) [82,83] solvent model was used in the above simulations.

The two-photon absorption cross section experimentally registered is described by the equation [84,85]

(6) σOF2=8π3α2η3er⋅ω2gωΓF2

where Γ*_F_* is the broadening of the final state (*F*) due to its finite lifetime, *α* is a fine structure constant, g(*ω*) provides the spectral line profile, which often is assumed to be a *δ*-function, *ω* is the frequency of absorbed photons, and $\langle \delta^{OF}\rangle$ is the two-photon transition probability for the transition from the *S_GS_* to a final state. In the case of a molecule absorbing two photons of the same energy in isotropic media, the degenerate $\langle \delta^{OF}\rangle$ in an isotropic medium using a linearly polarized laser beam given by [86]:

(7) $$\langle \delta^{OF}\rangle =\frac{1}{15}\underset{ij}{\sum }\left[S_{ii}^{OF}{\left(S_{jj}^{OF}\right)}^{*}+2S_{ij}^{OF}{\left(S_{ij}^{OF}\right)}^{*}\right]$$

In this equation, $S_{ij}^{OF}$ is the second-order transition moment given by:

(8) $$S_{ij}^{OF}=\frac{1}{\eta }\underset{k}{\sum }\left[\frac{\langle 0\left|\zeta_{1}\cdot \mu_{i}\right|K\rangle \langle K\left|\zeta_{2}\cdot \mu_{j}\right|F\rangle }{\omega_{\alpha }-\omega_{1}}+\frac{\langle 0\left|\zeta_{2}\cdot \mu_{i}\right|K\rangle \langle K\left|\zeta_{1}\cdot \mu_{j}\right|F\rangle }{\omega_{\alpha }-\omega_{2}}\right]$$

where $\eta \omega_{1}+\eta \omega_{2}$ should satisfy the resonance condition and $\langle 0\left|\zeta_{1}\cdot \mu_{i}\right|K\rangle$ stands for the transition moment between electronic states |0> and |*K*>, respectively. ζ is the vector defining polarization of photons.

The quadratic response functions formalism [87,88] and DALTON 2011 program [89,90] were used in TPA calculations with the self-consistent reaction field (SCRF) as a solvent model and CAM-B3LYP/6-311++G(d,p).

The binding properties of tested derivatives were studied by performing a series of AutoDock 4.2 [91,92,93] simulations. The Concanavalin A [94] and Human Serum Albuminum (HAS) [95] were used as ligands representing the protein. The grid box, with a grid spacing of 1 Å and the dimensions of 16 Å, was adjusted in such way that the space included the individual NH_2_ group of lysine’s side chain, in subsequent simulations. The docking region on the target protein was defined by establishing a cubic grid box with the dimensions of 16 Å and a grid spacing of 1 Å. In simulations, the Lamarkian genetic algorithm was employed to identify the appropriate binding energy and conformation of compounds. The investigation of the binding site was performed ten times for each lysine using a united-atom scoring function implemented in AutoDock Vina [96].

The biological activities were simulated using a combination of the 3D/4D QSAR BiS/MC and CoCon algorithms [97,98,99]. The first algorithm performs the restricted docking of compounds to receptor pockets. The second determines the relationships between the bioactivity and the parameters of interactions in the “receptor-ligand” complexes for estimating hydrogen bond energies. BiS/MC aligns compounds onto each other by considering their fields as being represented by van der Waals ($\varphi_{m}^{VDW}$) and Coulomb ($\varphi_{m}^{q}$) potentials at point on the molecular surface:

(9) $$\varphi_{m}^{q}=\overset{N}{\underset{i=1}{\sum }}\frac{q_{i}}{R_{im}}c\;\;\;\;\;$$

(10) $$\varphi_{m}^{VDW}=-2\overset{N}{\underset{i=1}{\sum }}V_{im}\frac{2^{3}r_{i}^{3}}{R_{im}^{6}}\;\;\;$$

where *N* is the total number of atoms in the molecule, *R_im_* is the distance from a point *m* to atom *i, q_i_* is the charge on the atomi, *V_im_* is the potential energy minimum of the Lennard–Jones equation of *i*th atom of the molecule, *c* is a scaling coefficient, *r_i_* is the van der Waals radius of atom. The receptor is represented as a set of pseudoatoms whose parameters can be calculated from the complementarity formalism:

(11) $$q_{m}=-\frac{\varphi_{m}^{q}}{\sum_{i=1}^{N}\frac{c}{R_{im}}\;\;}$$

(12) $$r_{m}=\sqrt[{3}]{\frac{\varphi_{m}^{VDW}}{-2^{3}\sum_{i=1}^{N}\frac{2V_{im}}{R_{im}^{6}}\;\;\;}}$$

where, *r*_m_ and *q*_m_ are the radius and charge of the pseudoatom located in the *m*th point. The compounds are oriented in the receptor model and the maximal total probability of the interaction of a compound with the model receptor is optimized. This is done using the Bis/MC and characteristics computed with MERA force field. The “receptor-ligand” complexes can be studied with CoCon approach for the determination of the mechanisms of the biological activity of molecules and for the search of active centers of receptors and ligands.

## 3. Results

### 3.1. Experimental Section

**Figure 1:**
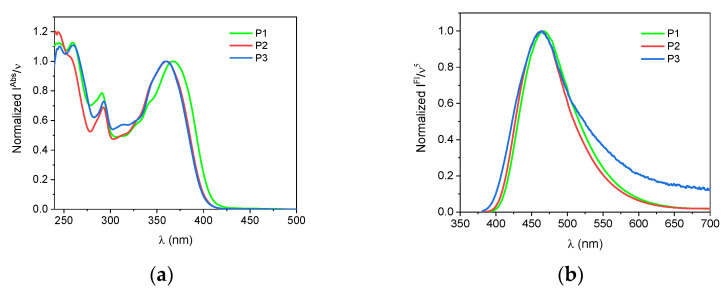
Scaled and normalized, steady-state absorption (left panel, **a**) and fluorescence (right panel, **b**) spectra of the studied compounds in CHCl_3_.

**Figure 2:**
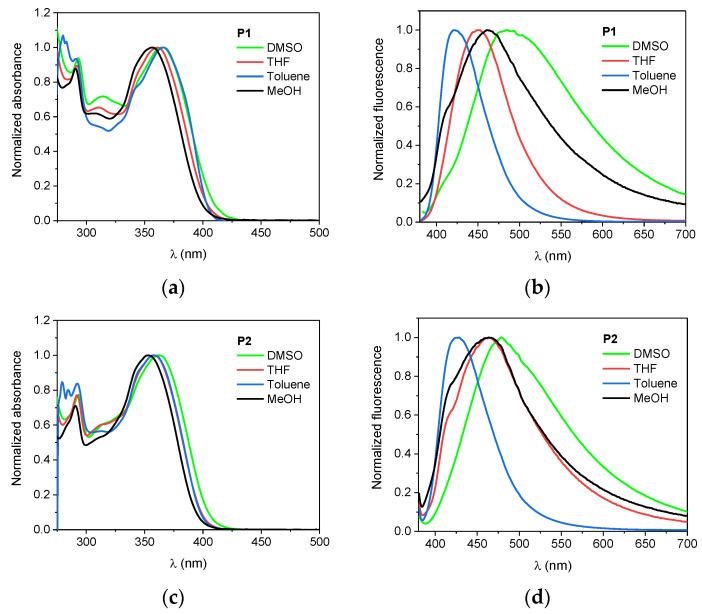

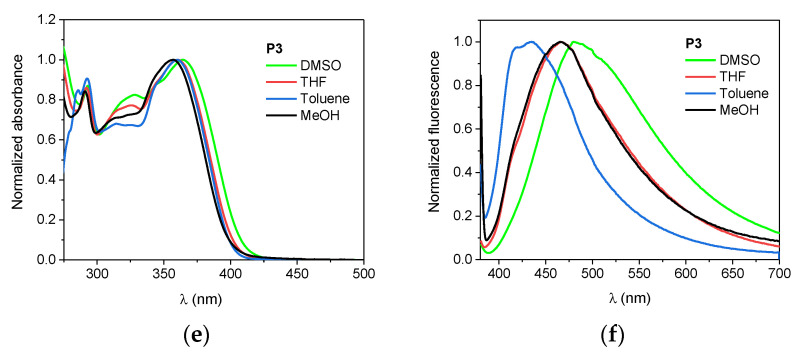
Normalized absorption (left panel: **a**,**c**,**e**) and fluorescence (right panel; *λ*_EX_ = 366 nm; **b**,**d**,**e**) spectra of the dyes in DMSO, THF, toluene, and MeOH.

The photophysical properties of carbazole dyes featuring two Cl substituents were studied in five organic solvents and compared with their structural analog containing one Cl atom at position 4 of the phenyl ring (Figure 1 and Figure 2, Table 1). The introduction of an additional chlorine atom to the molecule, regardless of its position, does not shift significantly both the absorption and fluorescence bands position (Figure 1). The shift in CHCl_3_ is ca. 8 nm and 5 nm for absorption and fluorescence spectra, respectively. Each of the dyes absorbed UV light with the most red-shifted absorption band of *π*→π* character in the range 320–400 nm. Another absorption band is below 300 nm. 

The longest wavelength absorption band is sensitive to solvent polarity, however, the bathochromic effect is not significant. The change of the solvents from toluene to DMSO causes only a few nanometer shift. These compounds afforded yellow solutions of different intensities whose molar extinction coefficients varied from 2.22 × 10^4^ M^−1^ cm^−1^ to 3.57 × 10^4^ M^−1^ cm^−1^ depending on the solvent used.

Concerning the tested compounds, the maximum fluorescence is largely affected by the solvent polarity with shift ca. 65 nm, 50 nm and 45 nm on going from toluene to DMSO for P1, P2, and P3, respectively. Furthermore, they characterize higher values of the Stokes’ shift in polar solvents than in non-polar ones. The effect is typical for compounds with different charge distribution in the excited state as compared to the ground state and suggests greater stabilization of the excited singlet state in a polar environment. Such behavior shows compounds with charge-transfer character for which excitation causes an increase in the dipole moment [100,101,102]. The conclusion was supported by theoretical calculations. The calculation revealed that the dipole moment for all tested dyes increase after photon absorption. The change of dipole moment Δ*μ*_CT_ depends on the solvent used and substituent in the phenyl ring, e.g., the dipole moment of P1 increases moderately upon excitation (ca. 4 D), whereas, for the P2, the Δ*μ*_CT_ values are the smallest (ca. 1–3 D). 

The compounds are not highly fluorescent (Table 1), the highest quantum yields were determined to be in chloroform with values 10.98%, 5.77%, and 0.41% for 4-Cl, 2,4-diCl, and P3, respectively. The introduction of the second Cl atom into the molecule quenches fluorescence intensity. The lowest *φ*_Fl_ values are observed of P3 derivative regardless of the solvent polarity. In other solvents, the fluorescence quantum yield of the aza-dyes is at least one order of magnitude lower. 

**Figure 3:**
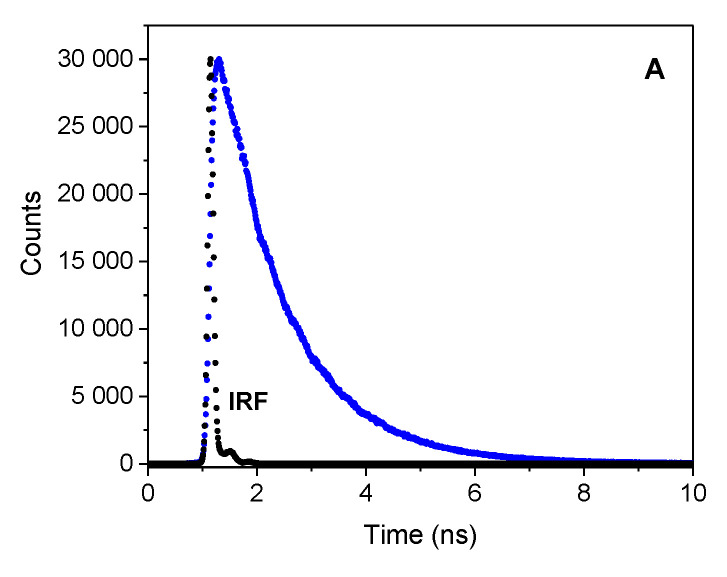

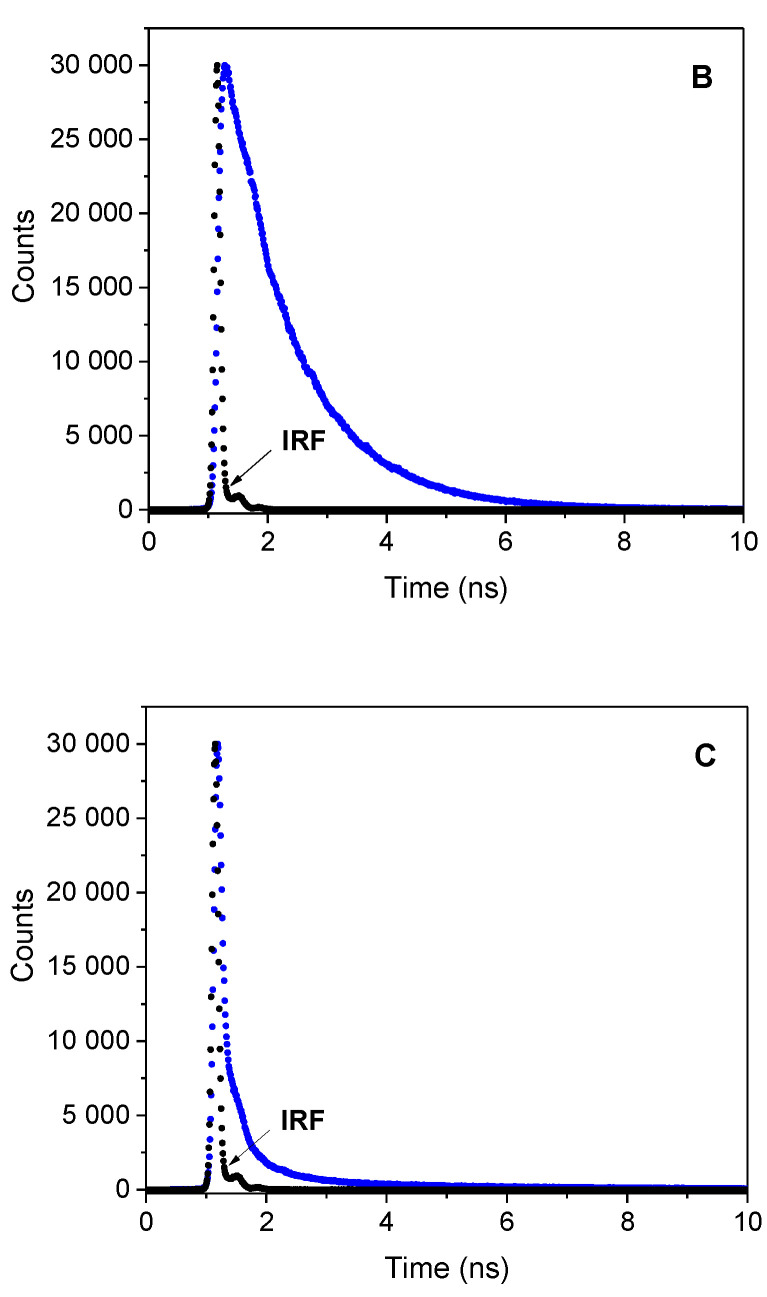
Fluorescence decay curve for (from top to bottom) P1 (**A**), P2 (**B**), and P3 (**C**), respectively, recorded in CHCl_3_; *λ_ex_* = 373 nm; *λ_em_* = 460 nm

Table 1 collects the fluorescence lifetimes obtained by deconvolution of decay curves (Figure 3) using, in most cases, the double-exponential fit. Analysis of the results indicates the contribution of the long (ca. 0.8–3.9 ns) and the short (ca. 74 ps–0.252 ns) lifetime components, with the share of the short-lived component being dominant in all solvents tested, as indicated by its amplitude. Presumably, the fluorescence from the non-relaxed excited state is related to the sort lifetime component, while the nanosecond fluorescence lifetime can be attributed to the relaxed state of CT character. The emission decay curves were well fitted with a mono-exponential function only in CHCl_3_. The determined fluorescence lifetimes were ca. 1.2 and 1.3 ns for P1 and P2 compounds, respectively.

Based on the fluorescence quantum yield and lifetime, the radiative *k_r_* and nonradiative *k_nr_* rate constants were calculated. The solvent polarity has a large effect on these values. The radiative rate constant are almost two order of magnitude lower than the nonradiative ones. Analyzing the ratio of the *k_nr_* vs. *k_r_*, the values of this parameter increase with increasing solvent polarity, e.g., from 8.1 in CHCl_3_ and 43 in toluene to 559.2 DMSO for P1 derivative. This ratio is even higher in methanol. The large *k_nr_* values for all dyes in polar aprotic and protic solvents indicate that the deactivation of the excited state occurs mainly by non-radiative channel.

### 3.2. Theoretical Sections

#### 3.2.1. Electrochemical Properties

**Figure 4:**
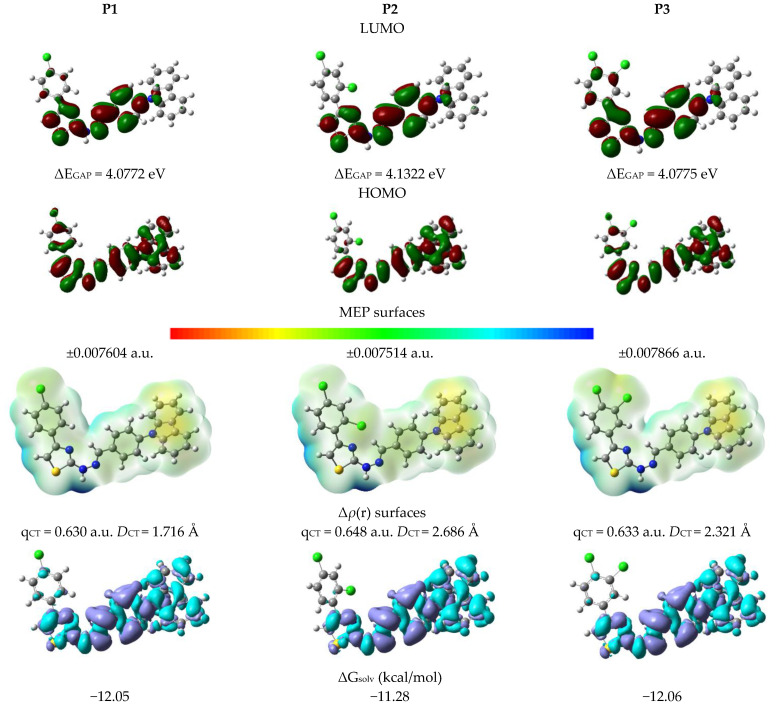
Physicochemical properties in water.

The charge-transfer (CT) excitation for the tested derivatives corresponds to the HOMO→LUMO transition (Figure 4). For P1, the HOMO electrons reach along the entire structure of the compound. The attachment of an additional chlorine atom leads to that this electron density practically disappear on the benzene ring. In turn, LUMO electrons are delocalized mainly on the thiazole ring, the *π*-electron bridge, and the benzene attached to the carbazole part. In this case, the presence of additional chlorine atoms does not change the position of this frontal orbital. The transfer of electrons towards the system of conjugated bonds and the thiazole ring indicates that the S_CT_ can be assigned as a *π*-π* transition mixed with an intramolecular charge-transfer (ICT) process. Considered below are the linear optical properties corresponding to the HOMO→LUMO photoexcitation. However, as further analysis will show, contributions from other orbitals cannot be ignored. From the data of the HOMO and LUMO energies, the parameters that indicate the chemical behavior of the studied molecules have been calculated as shown in Table S1. The results show that the energy separation between HOMO-LUMO orbitals of the studied compounds changes slightly. In the gas phase, the lowest value of ΔE_GAP_ is characteristic for P3 (3.9531 eV), while the highest value for P2 (4.0350 eV), and the difference between them is 0.0819 eV. As the polarity of the solvent increases, a monotonous increase in ΔE_GAP_ is observed, reaching its maximum values in the aqueous phase, 4.0772 eV, 4.1322 eV, and 4.0775 for P1, P2, and P3 respectively. On this basis, it is necessary to access the influence and molecular position of chlorine atoms on the energy gap. The derivative with two atoms attached in the 2,4 position has the highest value of ΔE_GAP_. Conversion of –Cl to position 3,4 reduces this value by an average of 0.7 eV. At the same time, derivative P3 is described with lower values of ΔE_GAP_ compared to monosubstituted, but only in GP and Toluene. A higher electronic chemical potential implies the absconding nature of an electron from an equilibrium system. The values of the chemical potential are negative in each case, which indicates a spontaneous interaction with other molecules. The electrophilicity indicates that P3 is the most and P2 the least electrophilic in nature among the studied systems. The tested dyes are characterized by low value of η and should be considered as soft compounds with very high reactivity. High *χ* value indicates an easy creation of covalent bonds during various biological and chemical processes. 

According to Figure 4, the most negative site (yellow and red regions, for electrophilic attack) is located on the pyrrole part of the carbazole. In turn, the most electropositive place (blue zones, for nucleophilic attack) is the nitrogen of the *π*-electron bridge with the attached hydrogen atom. In this case, the number and location of connection of chlorine substituents does not affect the location of sites for nucleophilic and electrophilic attack. However, this change affects the value of the accumulated positive and negative charge. The presence of two chlorine substituents results in both the highest and the lowest value of the accumulated charge. Against this background, it should be assumed that the discussed derivatives will not show a tendency to form hydrogen bonds as *H-*donor. However, the formation of a hydrogen bond can be considered as the consequence of the electrostatic potentials.

The plots of density variation upon photoexcitation (Δ*ρ*(r), Figure 4) show that the density depletion zones (blue) are mostly delocalized on the thiazole ring, the *π*-electron linker and the carbazole. In turn, the regions of density increment (purple) in any case, do not appear on the carbazole part and the outer benzene ring. The polarity of the environment affects the parameters describing Δ*ρ*(r) (Table S2). The *q_CT_* decreases monotonously as a function of solvent polarity. The largest difference Δ*q_CT_* between the extreme media, i.e., gas and water phase, is characteristic for P3, amounting to 0.217 e. At the same time, this derivative is characterized by the highest value of *q_CT_* in vacuum, while in the polar environments it is P2. The obtained values show slight differences between P1 and P3, which are between 0.002 e and 0.003 e. For each derivative, *D*_CT_ is reduced with increasing solvent polarity and the difference Δ*D_CT_* is 0.185 Å, 0.198 Å, and 0.217 Å for P1, P2, and P3, respectively. The charge-transfer distance depends on the number and the place of the attached chlorine atoms. In each analyzed medium, P1 is characterized by the lowest *D_CT_* value. In the case of the disubstituted derivatives, in the gas phase and toluene, P3 has the highest *D*_CT_ values while P2 the lowest. Starting with THF, the opposite relationship is observed. For all molecules, the Δ*ρ*(r) analysis indicates that the contributions from HOMO-LUMO transitions are major but minor (HOMO→LUMO+1 contribution of 3%) from other orbitals should be expected.

The free energy of solvation (Δ*G_solv_)* analysis (Table 2 and Table S3) showed, that all derivatives are well soluble in the tested media and the Δ*G_solv_* is lower than −20 kcal/mol. However, strongly polar solvents (transition from DMSO to water) may slightly increase the Δ*G_solv_* value).

#### 3.2.2. Linear and Nonlinear Optical Properties

The calculated absorption maxima ($\lambda_{max}^{Ab}$) determined for investigated compounds are presented in Table 2 and Table S4. When referring to the experimental values, the LC-wPBE functional is characterized by the greatest deviation (hypsochromic shift), as the mean absolute error is 62.44 nm. The hypsochromic shift is also characteristic for CAM-B3LYP, for which the error of determination is classified at the level of 36.42 nm. Bathochromic shifts are observed for the remaining functionals. However, it should be emphasized that the smallest deviation is obtained with PBE0, for which the average error is 5.20 nm and 4.53 nm for the cLR and vertical, respectively. For carbazole derivatives, the good agreement between the measured and theoretical data with the use of this functional has also been described earlier [103], as well as for the other classes of compounds. In particular, this functional approximates very accurately the optical properties of organic compounds in combination with the 6-311++G(d,p) basis set [104,105,106,107,108,109,110,111]. The use of PBE0 in conjunction with this functional base leads to an error in relation to the experimental values at a level not exceeding 5%. The popular B3LYP functional has been omitted from this study. It describes spectroscopic parameters with a high error of determination in relation to the measured values, even up to 17% [112]. On the other hand, when comparing the vertical values with those determined within the cLR approximation, the average difference between them is only 0.50 nm, while the largest deviations are observed in toluene and with the increase of the environment polarity this difference decreases. As for the measured values, the position of the maximum absorption band depends on the polarity of the solvent. Considering only the values obtained using PBE0, for P1 and P3 non-monotonous behavior is observed as a function of the medium polarity, for vertical, cLR, and experimentally measured values. For P2, the theoretical values indicate a practically monotonous increase in the excitation energy with an increase in the environment polarity. However, in the experimental values, the behavior is not monotonous. The position of $\lambda_{max}^{Ab}$ is also influenced by the presence of chloro substituents. Taking into account the increasing values of $\lambda_{max}^{Ab}$, the tested derivatives can be arranged in the vacuum as P2→P1→P3. In the water phase, there is a disturbance of this series: P2→P3→P1. All these observations together with MEP analysis, prove the possibility of occurrence of specific interactions of tested derivatives with solvent molecules. This is due to the fact that in polar medium a better stabilization and larger polarization and of the S_GS_ occurs. In turn, this relationship increases the excitation energy. Similar dependencies were presented by the Adhikari group [113]. This observation is not in accordance with the polarity of the S_CT_ ($\Delta \mu_{CT-GS}$) (Table 2 and Table S5). Firstly, all tested derivatives are characterized by the *μ_CT_* > *μ_GS_* relationship in each solution. This is characteristic of the presence of positive solvatochromism. Moreover, *μ_GS_* values increase monotonously as a function of environmental polarity. In the case of *μ_CT_*, for P1 non-monotonous behavior is observed under the influence of increasing solvent polarity, and the value in the vacuum is greater than the value in the aqua phase by 0.71 D. For the remaining derivatives, a monotonous decrease in μ_CT_ is observed. The most polar excited state ($\Delta \mu_{CT-GS}$) in slightly polar environments is characteristic for P3, and in medium and strongly polar environments-P1. It can also be noticed that the presence of an additional chlorine atom lowers the $\Delta \mu_{CT-GS}$ value. In water, the polarity of the excited state of P2 is lower than P1 by 3.20 D, and in relation to P3 by 0.78 D. For all derivatives, $\Delta \mu_{CT-GS}$ decreases monotonously in the function of medium polarity. In general, the lowest-lying excited state of the tested molecules is a relatively weak polar state. Therefore, the pure electrostatic contributions to the solvent–solute interactions should not occur. However, short-range specific interactions, e.g., self-aggregation and *H*-bond may be present. Similar solvatochromic effects have been demonstrated in studies on the 3,6-bis-((*N*-ethylcarbazole-3-)-propene-1-keto)-N-ethylcarbazole [114]. In turn, Bingul at al. [115] exploring the (6-ethyl-1,6-dihydropyrrolo [3,2-c]carbazol-2-yl)metanol and (6-ethyl-6,11-dihydro-1H-dipyrrolo[3,2-c:2′,3′-g]carbazole-2,10-diyl)dimethanol showed, that oxochromic groups such as –OH and –NH in the structure of compound increase the self-aggregation in solvents and this causes the $\lambda_{max}^{Ab}$ shift to the red wavelength. Examining the solvatochromic effect carbazole-based dendrimers indicates the dendrimers possess ICT from the carbazole (donor, D) to the ehynyl (acceptor, A) units. The 9-(4-(2-(3-(2-(4-(9*H*-carbazol-9-yl)phenyl) ethynyl)phenyl) ethynyl)phenyl)-9*H*-carbazole and 3,6-di-*tert*-butyl-9-(4-(2-(3-(2-(4-(3,6-di-*tert*-butyl-9*H*-carbazol-9-yl)phenyl)ethynyl)phenyl) ethynyl)phenyl)-9*H*-carbazole have higher solvatochromic displacements than 3,6-di-*tert*-butyl-9-(4-(2-phenylethynyl)phenyl)-9*H*-carbazol mainly due to the presence an additional D/A unit. Moreover, although the *meta*- and *para*-substituted didendrons also have an additional D/A group, it shows less solvatochromic shift [116]. Furthermore, photophysical and electrical properties of 2,6-polyphenylquinolines containing an oxygen or phenylamine bridging group have shown that the photophysical parameters in solvents increase with the phenylamine bridging unit in place of the oxygen one and the using of indolocarbazole instead of carbazole [117].

Table 2 and Table S6 present the values of the de-excitation energy ($\lambda_{max}^{Fl}$). For determining this property, only the PBE0 functional was used, as it best describes the optical properties of the tested derivatives. Its use leads to values with a mean absolute error of 5.18 nm and 3.41 nm for cLR and vertical values, respectively. The use of approximation of the cLR shifts the fluorescence maximum towards longer wavelengths, on average by 1.64 nm. As for the absorption bands, the position of $\lambda_{max}^{Fl}$ is sensitive to solvent changes and its monotonic growth behavior is observed. This relationship is consistent with the data obtained experimentally. This confirms that the tested derivatives have neutral S_CT_ and charge-separated S_GS_ as a result of interactions with solvent molecules. The attachment of a second chlorine atom lowers the gas phase de-excitation energy, and the fluorescence peak shifts towards longer wavelengths. Moreover, the position of attached Cl atom shifts the $\lambda_{max}^{Fl}$ maximum. Changing the position from 4 to 3 causes a bathochromic shift by another 7.89 nm. At the same time, the analyzed derivatives can be arranged in the following way: P1→P2→P3. The introduction of molecules into solutions disrupts this ranking. In toluene and THF, the molecules form a series analogous to the vacuum. They form the P2→P3→P1 relationship in CH_3_Cl and water, and P2→P1→P3 in MeOH.

The presented carbazole derivatives are characterized by relatively high Stokes’ shift values ($\Delta \nu^{St}$). Its value for all molecules increases monotonously in the solvent polarity function. For the gas-water phase transition, $\Delta \nu^{St}$ changes from 3105.90 cm^−1^ to 7057.13 cm^−1^, 3738.85 cm^−1^ to 6867.71 cm^−1^, and 3753.66 cm^−1^ to 6665.81 cm^−1^ for P1, P2, and P3, respectively. Based on this, it can be clearly stated that newly synthesized carbazole derivatives meet all requirements for fluorescent markers.

Recently, the subject of research of many scientific centers is the analysis of the value of polarizability (〈α〉) and the first hyperpolarizability (*β*_vec_) of molecules exposed to intense laser light. This approach helps to understand many linear and NLO properties. Theoretically determined NLO values are shown in Table 2 and Table S7. For each derivative, 〈α〉 values increase with increasing solvent polarity. At the same time, chlorine disubstituted compounds show a higher level of NLO response. Furthermore, the Δ〈α〉 difference in the gas phase between P2/P3 and P1 exceeds 500 a.u., and decreases as a function of the medium polarity, assuming the lowest value in the water phase not exceeding 22 a.u. The influence of the place of attachment of the chlorine atom is also noted. The 3,4-dichloro compound is described by slightly higher 〈α〉 values. In the case of the first hyperpolarizability, for each molecule, a decrease in its value with an increase in the environment polarity is observed, and the difference between vacuum and DMSO, as the most extreme values, is 618.18 a.u., 787.05 a.u., and 817.69 a.u. for P1, P2, and P3, respectively. At the same time, despite the monotonous decrease in *β*_vec_ values, the change DMSO to the aqua phase is accompanied by its increase, on average by 36.78 a.u. Based on this, it should be assumed that the presence of two chlorine atoms, and in particular one attached in the 3-position, will maximize the nonlinear response.

Table 2 and Table S8 show the TPA values based on the CAM-B3LYP functional. For all compounds, both the values expressed in a.u. and in GM decrease monotonously as a function of solvent polarity. The calculated values of $\langle \delta^{OF}\rangle$ and $\sigma_{OF}^{\left(2\right)}$ are relatively low and indicate the limitation of the usefulness of the tested molecules in studies with the use of two-photon absorption. However, similar to 〈α〉 and *β*_vec_, the presence of two chlorine substituents is a factor in maximizing the TPA value.

#### 3.2.3. Biological Properties

**Figure 5:**
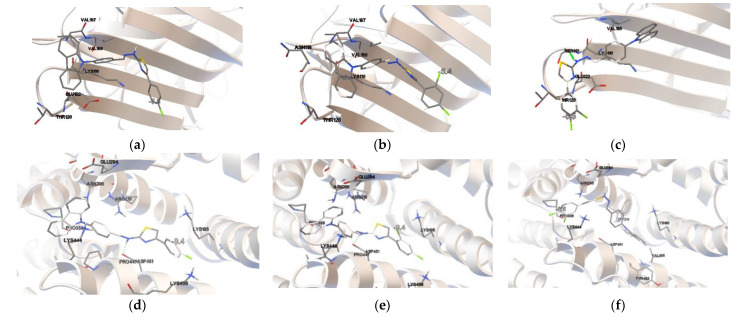
The results of Auto Dock simulations. (**a**) P1-ConA; (**b**) P2-ConA; (**c**) P3-ConA; (**d**) P1-HAS; (**e**) P2-HAS; (**f**) P3-HSA

The presented studies showed that the analyzed dyes could not be used in the studies taking into account the two-photon absorption. However, by introducing an appropriate reactive group interacting with the protein into their structure, a valuable tool for single-photon visualization in bioimaging can be achieved. After the introduction of an appropriate reactive group [118], conjugation of the carbazole derivatives can take place with either lysines or cysteines. Concanavalin A (ConA) and Human Serum Albumin (HAS) were selected for this study. As shown in Figure 5, the number and place of attached chlorine atom does not affect the site of active interaction with the biomolecule. For ConA that site is LYS116. P1 and P3 have the highest affinity for this active center. In this site, the binding energy (Δ*G_b_*) is −5.5 kcal/mol (Table S9) and inhibition constant (K*_i_*) is 1.19 μM and 1.05 μM, respectively. P1 molecule is inserted into the aromatic cage formed by LYS116, VAL187, GLU122, VAL188, and THR120. The biocomplex is not stabilized by the presence of a *H*-bond and no π-π* interactions occur. For P3, the active space is created by LYS116, VAL188, SER117, GLU122, and THR122. The molecule as a potential fluorescent marker interacts in this zone with SER117, creating H-bond between the oxygen of the amino acid and the hydrogen of the bonded with nitrogen of *π*-electron bridge. For P2, the affinity to ConA slightly decreases and the molecule is surrounded by ASN118, VAL187, VAL188, LYS116, and THR120. As for the molecule monosubstituted with a chlorine atom, there are no additional interactions stabilizing the biosystem. During the interaction with HSA, the active site for the tested carbazole derivatives is CYS448. In the process of spatial adjusting, the dyes are surrounded by more amino acids. In this case, P1 and P2 have the highest affinity (Table S10), for which Δ*G_b_* is −9.4 kcal/mol, and K*_i_* is 0.75 μM and 0.89 μM, respectively. Both compounds are additionally stabilized by forming a hydrogen bond with the ASP451 oxygen. For P2, Δ*G_b_* is −8.6 kcal/mol and the system is also not stabilized by additional interactions in the dye-amino acid system.

P2 and P3 are characterized by the highest value of LogP amounting to 8.85. The lowest LogP was determined for P1, which is 8.29. According to the Lipiński rule [119], the LogP for pharmacologically active substances should not exceed 5. However, this rule was originally conceived to aid the development of orally bioavailable drugs [120]. For this reason, experimental studies to determine the bioavailability with different routes of administration of these derivatives should be performed. On this basis, the analyzed compounds should be characterized by very good permeability through cell membranes, which is a very valuable property in drug design. The calculated LogBCF in the range from −5.43 (P1) to −5.79 (P2 and P3) indicates the lack of bioaccumulation in the cells and tissues of living organisms, and the ease of excretion with urine. After fulfilling their optical role, all tested molecules should not bioaccumulate. The tested dyes are characterized by high metabolism by CYP450-2D6P1 (probability > 60%) and CYP450-2A4 (probability > 96%) (Table S11). This indicates the ease of excretion from the cells and tissues without interaction with other pharmacologically active substances and biomolecules.

The tested compounds should be treated as compounds that are relatively safe for the human body. The calculated LD50 value for an intraperitoneal route of administration is 363.80 mg/kg, 447.80 mg/kg, and 403.70 mg/kg for P1, P2, and P3, respectively. For an intravenous route of administration, the LD50 values are 31.34 mg/kg, 34.91 mg/kg, and 34.77 mg/kg; for an oral route of administration 699.00 mg/kg, 988.20 mg/kg, and 900.60 mg/kg and for a subcutaneous route of administration 362.80 mg/kg, 500.40 mg/kg, and 433.90 mg/kg. All analyzed carbazole derivatives show hepatotoxicity at the level of 57%. The number and place of attachment of chlorine substituents also does not change the mutagenicity, which is 58%. All molecules showed no carcinogenicity (59%), immunotoxicity (71–79%), and cytotoxicity (71–74%).

The investigated carbazole dyes are characterized by other biological activities, which allow them to be used in various other fields of medicine (Table S11). Firstly, regardless of the number and place of attachment of chlorine atoms, the tested compounds are characterized by a high probability of the following biological properties: Anti-Awesky Disease activity, anti-herpes simplex virus activity, anti-infectious laryngotracheitis activity, anti-rift valley fever activity, anti-adenovirus activity, anti-arrhythmic activity, anti-influenza activity Hong-Kong virus, anti-influenza A activity, anti-influenza B activity, anti-psychotic activity diazepine site, anti-tumor cycline-dependent kinase 4 inhibitory activity, anti-tumor topoisomerase II inhibitory activity, gamma-radioprotector activity mechanism I, human factor XA inhibitory activity, LOX inhibitory activity, progestagenic activity, and tuberculostatic dihydrofolate reductase inhibitory activity. At the same time, it is possible to notice the presence of some features depending on the presence of chlorine atoms. The 4-chloro and 3,4-dichloro derivatives are described by: analgetic activity (*p* > 84%), anti-tumor DNA anti-metabolitic activity (*p* > 67%), and gamma-radioprotector activity mechanism II (*p* > 69%). The 2,4-dichloro derivatives shows the following properties: anti-bacterial activity (*p* > 45%) and anti-tumor topoisomerase II inhibitory activity (*p* > 45%). Moreover, P1 and P2 exhibit alpha-radioprotector activity (*p* > 55%) and only 4-chloro derivative is characterized by vasorelaxant activity (*p* > 48%).

## 4. Conclusions

The work presents new carbazole derivatives with potential application in biochemistry and as active NLO substances. The performed analysis showed the environment effect on the $\lambda_{max}^{Abs}$ and $\lambda_{max}^{Fl}$ position of tested carbazole derivatives, which shows a non-monotonous behavior. The lack of monotonicity of the increase or decrease in the excitation and de-excitation energy values is more evident in the values determined experimentally than those obtained as part of the TD-DFT and cLR approximation. At the same time, the presented values of the μ_GS_ and μ_CT_ indicate that the newly synthesized derivatives should be characterized by positive solvatochromism. The influence of the number and point of attachment of chlorine substituents on optical properties is also presented. In the vacuum and in weakly polar environments, the absorption and fluorescence maxima are shifted batochromically with the change of the structure of the molecule from 4-Cl, through 2,4-diCl to 3,4-diCl. The transition to a more polar environment disrupts this dependence, and the bathochromic and hypsochromic shifts under the influence of structural changes cannot be clearly arranged and remain an individual feature of a given molecule. The tested derivatives are characterized by low fluorescence quantum yield and introduction of the second Cl atom into the molecule may quench fluorescence intensity. Therefore, before their further application, simulations should be carried out to improve this property by introducing additional groups. However, the relatively good bioavailability suggests the newly synthesized carbazole derivatives are good precursors for further pharmacological and medical studies. The theoretical and experimental values indicate the ICT character of the investigated derivatives, which increases in the Stokes’ shift in a more polar environment. The MEP analysis shows that *H*-bonds may form in the solute-protic solvent system. In the case of aprotic-polar environments, the tested dyes may create dipole–dipole interaction with solvent molecules. The calculated density variation upon photoexcitation confirms the CT character and the contributions from HOMO→LUMO transition and minor contributions from HOMO→LUMO + 1. The analysis of polarization and first hyperpolarizability showed the possibility of using the analyzed carbazole derivatives in nonlinear optics, such as in Second Harmonic Generation (SHG). At the same time, it was shown that the presence of two chlorine substituents, and in particular one attached in the 3-position, will maximize the nonlinear response. Moreover, the obtained values of TPA excluded the studied molecules for use in two-photon imaging.

Summarizing the biological results, it can be stated that the tested derivatives are good precursors for applications in in vitro and in vivo studies. The molecules show high affinity to proteins. The simulations carried out with the use of ConA and HSA showed that the number and place of attachment of the chlorine atom did not affect the site of active protein binding. Moreover, the binding free energies is not significantly dependent on the discussed structural changes. Thus, the introduction of an additional reactive group into the system, such as -CHO, -NCS, will allow the use of the presented derivatives as fluorescent probes in medical imaging. Furthermore, the molecules presented in this paper have many features that enable their use as valuable pharmaceutical preparations.

Toxicity studies showed that the tested carbazole derivatives should be treated as compounds that are relatively safe for the human body and an increased number of chlorine substituents tends to reduce their toxicity. Although these molecules do not exhibit carcinogenicity, immunotoxicity, and cytotoxicity, they have slight hepatotoxicity and mutagenicity characteristics. These properties should not affect their use in medical imaging, but may affect their full use in developing new drugs.

All the above means that the newly synthesized carbazole derivatives are excellent molecular structures for further pharmacological and medical studies.

## Figures and Tables

**Table 1:** Main photophysical parameters ^a^ for tested dyes.

Solvent	$\lambda_{max}^{Ab}$	*ε*	$\lambda_{max}^{Fl}$	∆*ν*	*φ* *_Fl_*	*τ* *_1_* *α* *_1_*	*τ* *_2_* *α* *_2_*	*χ* ^2^	*τ* *_av_*	*k_r_*	*k_nr_*	*k_nr_/k_r_*
	P1											
DMSO	365	2.50	489.4	6964	0.18	0.086 95.54	1.735 4.46	1.353	0.161	1.11	6.20	559.2
THF	361.5	2.55	456.8	5771	0.14	0.667 67.49	3.962 32.51	1.465	1.740	0.805	0.575	713.3
CHCl_3_	366	2.41	454.2	5306	10.98	-	1.261 100	1.579	1.261	8.71	0.71	8.1
Toluene	365.5	-	424.6	3808	2.27	0.226 99.22	0.777 0.78	1.364	0.230	9.86	4.24	43.0
MeOH	356.5	2.22	466.4	6610	0.13	0.11 79.27	1.188 20.73	1.403	0.333	0.375	3.00	798.7
-	P2											
DMSO	362.5	2.87	478.4	6683	0.19	0.074 95.94	1.56 4.06	1.481	0.134	1.42	7.43	524.8
THF	357.5	3.17	463.6	6402	0.12	0.103 71.41	2.864 28.59	1.4	0.892	0.13	1.12	854.9
CHCl_3_	359	2.90	448.6	5564	5.77	-	1.178 100	1.753	1.178	4.90	0.80	16.3
Toluene	357	2.90	428	4647	0.18	0.252 95.65	1.003 4.35	1.368	0.285	0.064	3.51	547.0
MeOH	353	3.53	463.6	6758	0.09	0.088 90.23	1.072 9.77	1.265	0.184	0.051	5.43	1061.8
-	P3											
DMSO	364	2.64	479.6	6622	0.19	0.074 96.23	1.633 3.77	1.581	0.132	1.47	7.55	513.8
THF	360.5	3.41	466.6	6308	0.17	0.131 41.66	3.917 58.34	1.327	2.340	0.073	0.43	582.7
CHCl_3_	358	3.07	449.4	5681	0.41	0.951 60.72	2.222 39.28	1.468	1.450	0.29	0.69	240.3
Toluene	360	3.02	434.6	4768	0.07	0.078 92.84	1.605 7.16	1.255	0.187	0.37	5.33	1432.6
MeOH	357	3.25	465	6506	0.15	0.097 84.19	1.051 15.81	1.418	0.248	0.598	4.03	673.5

^a^ fluorescence quantum yield (*φ_Fl_;* %), fluorescence lifetime (*τ*; ns), its amplitude (*α*) and correlation coefficient (*χ*^2^ ), ^2^ average fluorescence lifetime (*τ*_av_; ns), radiative (*k_r_*; 10^7^ s^−1^) and non-radiative (*k_nr_*; 10^9^ s^−1^) rate constants for tested compounds.

**Table 2:** Theoretical linear and nonlinear optical properties.

	Gas Phase			DMSO		
	P1	P2	P3	P1	P2	P3
$\lambda_{max}^{Ab}$(vert) ^1^	365.58	364.17	369.84	365.17	361.39	364.86
$\lambda_{max}^{Ab}$(cLR) ^1^	-	-	-	363.79	361.96	364.50
$\lambda_{max}^{Fl}$(vert) ^1^	413.68	421.57	429.46	490.12	479.26	480.51
$\lambda_{max}^{Fl}$(cLR) ^1^	-	-	-	492.16	480.46	481.60
$\langle \delta^{OF}\rangle$ ^2^	985.99	1493.83	1695.00	948.06	1435.18	1639.17
$\sigma_{OF}^{\left(2\right)}$ ^3^	4.37	6.72	7.51	4.18	6.43	7.23
*μ*_GS_ ^4^	3.60	4.17	4.85	5.17	5.90	7.12
*μ*_CT_ ^4^	10.01	10.69	11.47	9.47	6.88	8.93
〈α〉 ^2^	441.55	449.99	453.48	600.15	614.43	620.46
Δα ^2^	289.85	295.23	312.61	304.12	322.71	371.64
*β* _vec_ ^2^	722.09	953.31	1036.64	103.91	166.26	218.95

^1^ [nm]; ^2^ [a.u.]; ^3^ [GM]; ^4^ [D].

## Data Availability

Data is contained within the article or supplementary materials.

## Supplementary Materials

The following are available online at https://www.mdpi.com/article/10.3390/ma14113091/s1, Table S1: The frontier orbital energies in selected solvents. All values are given in eV, Table S2: CT parameters for the bright low-lying excited state, Table S3: Free energies (Δ*G*_solv_, kcal/mol) of solvation, Table S4: The theoretical vertical and cLR corrected excitation energies in nm. Table S5: Calculated values of dipole moments (in D) for the ground and CT excited state. Table S6: The theoretical de-excitation energies in nm determined using PBE0 functional. Table S7: Nonlinear optical properties. All values are given in (a.u.). Table S8: Two-photon absorption cross section. Table S9: Binding free energies (Δ*G_b_*, kcal/mol) obtained during AutoDock simulations with Conconavalin A. Table S10: Binding free energies (Δ*G_b_*, kcal/mol) obtained during AutoDock simulations with Human Serum Albuminum. Table S11: The calculated biological activities.

## Abbreviations

ConA	concanavalin A
HAS	Human Serum Albumin
GP	gas phase
CH_3_Cl	Chloroform
THF	TetraHydroFuran
MeOH	Methanol
DMSO	DiMethylSulfoxide
