# Supplementary material for: Effect of the Chloro-Substitution on Electrochemical and Optical Properties of New Carbazole Dyes
Source: Materials (Basel). 2021 Jun 4;14(11):3091. doi: 10.3390/ma14113091 (PMC8200205; doi:10.3390/ma14113091)
Supplement: Supplementary file 1 [file materials-14-03091-s001.zip › materials-1223975-supplementary.pdf]

materials

Supplementary materials

Effect of the Chloro-Substitution on Electrochemical and Optical Properties of New Carbazole Dyes

Przemysław Krawczyk [1,*], Beata Jędrzejewska [2], Klaudia Seklecka [3], Joanna Cytarska [3] and Krzysztof Z. Łączkowski [3]

[1] Department of Physical Chemistry, Faculty of Pharmacy, Collegium Medicum, Nicolaus Copernicus University, Kurpińskiego 5, 85-950 Bydgoszcz, Poland
[2] Faculty of Chemical Technology and Engineering, UTP University of Science and Technology, Seminaryjna 3, 85-326 Bydgoszcz, Poland; beata@utp.edu.pl
[3] Department of Chemical Technology and Pharmaceuticals, Faculty of Pharmacy, Collegium Medicum, Nicolaus Copernicus University, Jurasza 2, 85-089 Bydgoszcz, imaginowa@o2.pl (K.S.); Poland; cytar@cm.umk.pl (J.C.); krzysztof.laczkowski@cm.umk.pl (K.Z.Ł.)
* Correspondence: przemekk@cm.umk.pl

Table S1. The frontier orbital energies in selected solvents. All values are given in eV.

Compound-solvent	E_{HOMO}	E_{LUMO}	ΔE_{GAP}	η	μ	χ	σ	*pi*	*S*	ω	ΔNmax
P1-GP	−5.7854	−1.7798	4.0056	2.0028	−3.7826	3.7826	0.4993	−3.7826	1.0014	3.572	1.8886
P1-Toluene	−5.8091	−1.7564	4.0527	2.0263	−3.7827	3.7827	0.4935	−3.7827	1.0132	3.5307	1.8668
P1-CH_3Cl	−5.8363	−1.7596	4.0766	2.0383	−3.798	3.798	0.4906	−3.798	1.0192	3.5383	1.8633
P1-THF	−5.8363	−1.7626	4.0736	2.0368	−3.7995	3.7995	0.491	−3.7995	1.0184	3.5437	1.8654
P1-MeOH	−5.8363	−1.7626	4.0736	2.0368	−3.7995	3.7995	0.491	−3.7995	1.0184	3.5437	1.8654
P1-DMSO	−5.8502	−1.7733	4.0769	2.0385	−3.8117	3.8117	0.4906	−3.8117	1.0192	3.5638	1.8699
P1-H_2O	−5.8515	−1.7743	4.0772	2.0386	−3.8129	3.8129	0.4905	−3.8129	1.0193	3.5658	1.8704
P2-GP	−5.756	−1.721	4.035	2.0175	−3.7385	3.7385	0.4957	−3.7385	1.0087	3.4638	1.853
P2-Toluene	−5.808	−1.7101	4.0979	2.0489	−3.759	3.759	0.4881	−3.759	1.0245	3.4482	1.8346
P2-CH_3Cl	−5.8428	−1.7237	4.1191	2.0595	−3.7833	3.7833	0.4855	−3.7833	1.0298	3.4748	1.8369
P2-THF	−5.8589	−1.7335	4.1253	2.0627	−3.7962	3.7962	0.4848	−3.7962	1.0313	3.4933	1.8404
P2-MeOH	−5.8589	−1.7335	4.1253	2.0627	−3.7962	3.7962	0.4848	−3.7962	1.0313	3.4933	1.8404
P2-DMSO	−5.8877	−1.7558	4.1319	2.0659	−3.8218	3.8218	0.484	−3.8218	1.033	3.5349	1.8499
P2-H_2O	−5.8902	−1.758	4.1322	2.0661	−3.8241	3.8241	0.484	−3.8241	1.033	3.539	1.8509
P3-GP	−5.7963	−1.8432	3.9531	1.9765	−3.8197	3.8197	0.5059	−3.8197	0.9883	3.6909	1.9325
P3-Toluene	−5.827	−1.8035	4.0236	2.0118	−3.8152	3.8152	0.4971	−3.8152	1.0059	3.6177	1.8964
P3-CH_3Cl	−5.8502	−1.7969	4.0532	2.0266	−3.8235	3.8235	0.4934	−3.8235	1.0133	3.6069	1.8867
P3-THF	−5.8608	−1.7972	4.0636	2.0318	−3.829	3.829	0.4922	−3.829	1.0159	3.6079	1.8845
P3-MeOH	−5.8608	−1.7972	4.0636	2.0318	−3.829	3.829	0.4922	−3.829	1.0159	3.6079	1.8845
P3-DMSO	−5.8785	−1.8018	4.0766	2.0383	−3.8401	3.8401	0.4906	−3.8401	1.0192	3.6174	1.884
P3-H_2O	−5.8798	−1.8024	4.0775	2.0387	−3.8411	3.8411	0.4905	−3.8411	1.0194	3.6184	1.8841

Table S2. CT parameters for the bright low-lying excited state.

Compound-solvent	q_{CT}	D_{CT}
P1-GP	0.815	3.541
P1-Toluene	0.692	2.841
P1-CH_3Cl	0.656	2.420
P1-THF	0.647	2.156
P1-MeOH	0.636	1.787
P1-DMSO	0.629	1.683
P1-H_2O	0.630	1.716
P2-GP	0.846	3.675

P2-Toluene	0.725	3.319
P2-CH_3Cl	0.684	3.099
P2-THF	0.668	2.971
P2-MeOH	0.652	2.751
P2-DMSO	0.646	2.637
P2-H_2O	0.648	2.686
P3-GP	0.850	3.847
P3-Toluene	0.720	3.446
P3-CH_3Cl	0.672	3.079
P3-THF	0.655	2.849
P3-MeOH	0.636	2.422
P3-DMSO	0.631	2.294
P3-H_2O	0.633	2.321

Table 3. Free energies (ΔG_{solv}, kcal/mol) of solvation.

Solvent	P1	P2	P3
Toluene	−24.80	−25.56	−20.94
CH_3Cl	−25.23	−26.36	−25.36
THF	−23.02	−24.04	−22.66
MeOH	−20.89	−22.66	−20.97
DMSO	−21.44	−20.68	−19.34
H_2O	−12.05	−11.28	−12.06

Table S4. The theoretical vertical and cLR corrected excitation energies in nm.

Compound-solvent	Vertical								*cLR*
	CAM-B3LYP		HSEH1PBE		LC-wPBE		PBE0		PBE0
Header	*λ*	*f*	*λ*	*f*	*λ*	*f*	*λ*	*f*	*λ*
P1-GP	318.62	1.0295	384.44	0.4833	292.00	1.1466	366.58	0.6332	-
P1-Toluene	325.05	1.1863	383.34	0.7308	298.40	1.2722	368.31	0.8900	369.94
P1-CH_3Cl	324.64	1.1777	380.26	0.7772	298.27	1.2572	366.27	0.9232	366.32
P1-THF	324.44	1.1718	379.00	0.7961	298.15	1.2497	365.45	0.9332	365.03
P1-MeOH	323.77	1.1580	376.94	0.8146	297.65	1.2336	363.97	0.9393	363.19
P1-DMSO	324.99	1.1760	378.01	0.8460	298.72	1.2504	365.17	0.9667	363.79
P1-H_2O	324.06	1.1593	376.99	0.8274	297.92	1.2337	364.14	0.9482	363.12
P2-GP	316.77	1.0429	382.03	0.4637	290.82	1.1670	364.17	0.6118	-
P2-Toluene	322.76	1.2253	379.60	0.7119	296.96	1.3108	364.73	0.8830	368.68
P2-CH_3Cl	322.53	1.2236	376.44	0.7626	297.04	1.3000	362.66	0.9258	364.96
P2-THF	322.36	1.2188	375.14	0.7818	296.99	1.2921	361.79	0.9396	363.43
P2-MeOH	321.88	1.2041	373.21	0.8008	296.67	1.2739	360.42	0.9494	361.32
P2-DMSO	322.97	1.2261	374.02	0.8332	297.66	1.2941	361.39	0.9801	361.96
P2-H_2O	322.08	1.2084	373.08	0.8127	296.90	1.2770	360.42	0.9596	361.15
P3-GP	319.17	0.9851	390.11	0.4035	292.12	1.1090	369.84	0.5685	-
P3-Toluene	324.87	1.1538	386.15	0.6393	298.16	1.2426	369.50	0.8255	367.76
P3-CH_3Cl	324.42	1.1487	381.97	0.7002	298.05	1.2295	366.84	0.8711	368.76
P3-THF	324.11	1.1425	380.18	0.7240	297.88	1.2207	365.67	0.8852	366.72
P3-MeOH	323.45	1.1280	377.55	0.7545	297.39	1.2032	363.92	0.8988	363.99
P3-DMSO	324.52	1.1479	378.32	0.7840	298.38	1.2223	364.86	0.9253	364.50
P3-H_2O	323.62	1.1301	377.32	0.7661	297.58	1.2047	363.86	0.9067	363.71

Table S5. Calculated values of dipole moments (in D) for the ground and CT excited state.

Solvent	P1		P2		P3	
	μ_{GS}	μ_{CT}	μ_{GS}	μ_{CT}	μ_{GS}	μ_{CT}
GP	3.60	10.01	4.17	10.69	4.85	11.47
Toluene	4.33	8.59	4.95	7.92	5.90	11.31
CH_3Cl	4.74	8.63	5.38	7.04	6.46	11.41

THF	4.90	8.78	5.57	6.93	6.71	10.40
MeOH	5.14	8.68	5.85	6.90	7.05	8.99
DMSO	5.17	9.47	5.90	6.88	7.12	8.93
H_2O	5.18	9.30	5.92	6.82	7.15	8.83

Table S6. The theoretical de-excitation energies in nm determined using PBE0 functional.

Compound-solvent	Vertical	*cLR*
P1-GP	413.68	-
P1-Toluene	426.23	428.79
P1-CH_3Cl	455.17	458.12
P1-THF	458.21	459.87
P1-MeOH	467.55	468.99
P1-DMSO	490.12	492.16
P1-H_2O	490.08	492.10
P2-GP	421.57	-
P2-Toluene	430.09	432.55
P2-CH_3Cl	449.79	451.89
P2-THF	465.07	466.62
P2-MeOH	465.36	467.23
P2-DMSO	479.26	480.46
P2-H_2O	478.98	480.03
P3-GP	429.46	-
P3-Toluene	436.02	438.07
P3-CH_3Cl	450.87	451.69
P3-THF	467.72	469.11
P3-MeOH	468.02	469.30
P3-DMSO	480.51	481.60
P3-H_2O	480.37	481.49

Table S7. Nonlinear optical properties. All values are given in (a.u.).

Solvent	**P1**		P2		P3	
	$\langle\alpha\rangle$	β_{vec}	$\langle\alpha\rangle$	β_{vec}	$\langle\alpha\rangle$	β_{vec}
GP	441.55	722.09	449.99	953.31	453.48	1036.64
Toluene	509.14	357.41	518.27	698.20	523.43	723.30
CH_3Cl	542.73	329.57	559.08	640.52	564.75	651.69
THF	555.29	311.12	578.89	517.27	562.15	584.55
MeOH	591.31	294.49	610.89	318.21	616.92	370.75
DMSO	600.15	103.91	614.43	166.26	620.46	218.95
H_2O	602.52	145.21	617.62	205.09	623.67	249.16

Table S8. Two-photon absorption cross section.

Compound-solvent	$\langle\delta^{0F}\rangle$ (a.u.)	$\sigma_{OF}^{(2)}$ (GM)
P1-GP	985.99	4.37
P1-Toluene	968.91	4.27
P1-CH_3Cl	960.62	4.24
P1-THF	955.66	4.21
P1-MeOH	950.59	4.19
P1-DMSO	948.06	4.18
P1-H_2O	948.06	4.18
P2-GP	1493.83	6.72
P2-Toluene	1466.05	6.56
P2-CH_3Cl	1453.36	5.61
P2-THF	1447.04	6.48

P2-MeOH	1435.18	6.43
P2-DMSO	1435.18	6.43
P2-H_2O	1435.18	6.43
P3-GP	1695.00	7.51
P3-Toluene	1667.02	7.35
P3-CH_3Cl	1653.98	7.29
P3-THF	1647.36	7.26
P3-MeOH	1639.40	7.23
P3-DMSO	1639.17	7.23
P3-H_2O	1639.17	7.23

Table S9. Binding free energies (ΔG_b, kcal/mol) obtained during AutoDock simulations with Conconavalin A.

LYS	P1	P2	P3
30	−3.5	−4.8	−3.5
35	−4.8	−4.8	−4.9
36	−4.7	−3.6	−5.1
39	−3.7	−5.3	−3.7
46	−5.3	−4.6	−5.4
59	−4.7	−3.5	−4.8
101	−5.2	−5.2	−5.4
114	−4.8	−4.9	−4.8
116	−5.5	−5.4	−5.5
135	−3.9	−3.9	−4.0
138	−3.8	−4.0	−3.7
200	−3.8	−3.8	−3.9
Ter	−3.6	−3.3	−3.6

Table S10. Binding free energies (ΔG_b, kcal/mol) obtained during AutoDock simulations with Human Serum Albuminum.

CYS	P1	P2	P3
34	−3.9	−3.9	−4.5
53	−4.1	−4.8	−4.6
62	−4.0	−3.8	−4.3
75	−5.8	−5.9	−6.0
90	−4.1	−3.8	−3.5
91	−5.2	−4.7	−5.3
101	−5.9	−4.1	−4.3
124	−5.5	−5.6	−5.7
168	−5.9	−5.6	−4.6
169	−4.7	−5.1	−4.9
177	−5.5	−5.7	−6.0
200	−4.8	−3.4	−3.1
245	−1.2	−1.1	−0.9
246	−5.2	−4.4	−4.0
253	−2.7	−2.6	−2.6
265	−2.9	−2.6	−1.2
278	−4.9	−4.4	−4.8
279	−5.4	−5.4	−4.7
289	−1.0	−3.5	−0.7
316	−6.5	−6.5	−6.5
360	−4.9	−4.8	−4.9
361	−5.7	−5.7	−5.8
369	−5.0	−5.1	−5.1
392	−1.5	−0.4	−1.6
437	−6.5	−6.5	−6.7

438	−6.5	−6.5	−6.5
448	−9.4	−9.4	−8.6
461	−1.1	−0.9	−1.2
476	−4.5	−4.8	−4.7
477	−1.7	−1.1	−5.9
487	−6.2	−6.3	−6.5
514	−5.4	−5.6	−5.7
558	−3.3	−3.2	−4.7
559	−4.2	−4.2	−3.3
567	−0.4	−0.4	−4.3

Table S11. The calculated biological activities.

**Biological Activity**	**Probability**		
	**P1**	P2	P3
Acyl-CoA-holesterol transferase inhibitory activity	0.0206	0.0300	0.0000
Adrenoreceptor inhibitory activity Anti-Hypertensive Activity	0.0000	0.0002	0.0047
Alpha-Radioprotector activity	0.5540	0.6911	0.0964
Alpha-R-receptor inhibitory activity	0.0000	0.0001	0.0001
Analgetic activity	0.8459	0.0029	0.9195
Anti Awesky Desease activity	0.6482	0.4009	0.6460
Anti Crymean Haemorrhagic Fever activity	0.0000	0.0000	0.0000
Anti Herpes Simplex virus activity	0.6063	0.6430	0.6536
Anti infectious laryngotracheitis activity	0.8918	0.9259	0.8174
Anti Issyk-Kul Haemorrhagic Fever activity	0.0016	0.0007	0.0042
Anti Karelian Fever activity	0.0001	0.0007	0.0085
Anti Rift valley Fever activity	0.9966	0.9935	0.9967
Anti-Adenovirus activity	0.9990	0.9986	0.9984
Anti-Arrhythmic activity	0.9318	0.9629	0.9695
Anti-Bacterial activity	0.0000	0.4567	0.0778
Anti-Encephalitic activity	0.0005	0.0004	0.0015
Anti-Inflammatory activity combined action	0.0005	0.1863	0.0035
Anti-Inflammatory activity in vivo oedema paw carrageenin	0.0000	0.0000	0.0000
Anti-Inflammatory activity in vivo peritonitis	0.0000	0.0000	0.0000
Anti-Inflammatory activity ks2 p38 MAP-kinase inhibitors	0.0000	0.0000	0.0000
Anti-Influenza activity Hong-Kong virus	0.9623	0.9399	0.9241
Anti-Influenza A activity	0.8018	0.8734	0.7992
Anti-Influenza B activity	0.9652	0.9692	0.9431
Anti-Influenza Birds activity	0.0001	0.0000	0.0000
Anti-Oxidant activity	0.0357	0.0067	0.0938
Anti-Psychotic activity diazepine site	0.9970	0.9933	0.9955
Anti-Tumor Alkylic activity	0.0058	0.1042	0.0205
Anti-Tumor Antimitotic activity	0.0373	0.0652	0.0446

Anti-Tumor Cycline-dependent kinase 4 inhibitory activity	0.8459	0.9736	0.8545
Anti-Tumor Dihydrofolate reductase inhibitory activity	0.0093	0.0037	0.0177
Anti-Tumor DNA anti-metabolitic activity	0.9201	0.1697	0.6734
Anti-Tumor Topoisomerase I inhibitory activity	0.0723	0.4509	0.0366
Anti-Tumor Topoisomerase II inhibitory activity	0.8105	0.9267	0.9633
COX1 inhibitory activity	0.0042	0.0000	0.4718
COX2 inhibitory activity	0.0000	0.0000	0.0000
Gamma-radioprotector activity mechanism I	0.6187	0.6903	0.5276
Gamma-Radioprotector activity mechanism II	0.7537	0.2341	0.6956
HIV1 -proteaze inhibitory activity	0.6056	0.6666	0.7491
HT51 A inhibitory activity	0.0807	0.7630	0.0140
Human factor XA Inhibitory activity	0.3470	0.8484	0.5084
LOX inhibitory activity	0.8444	0.9422	0.8259
Progestagenic activity	0.2548	0.9987	0.9166
Tuberculostatic Dihydrofolate reductase inhibitory activity	0.9378	0.9927	0.9763
Tuberculostatics combined action	0.0007	0.0087	0.0083
Vasorelaxant activity	0.4894	0.0989	0.1084
Metabolism at CYP450 2D63c	0.9618	0.9832	0.9948
Metabolism at CYP450 3A4	0.5981	0.9987	0.6698
